# Glutamine synthetase expression rescues human dendritic cell survival in a glutamine-deprived environment

**DOI:** 10.3389/fonc.2023.1120194

**Published:** 2023-01-19

**Authors:** Robert Schoeppe, Nathalie Babl, Sonja-Maria Decking, Gabriele Schönhammer, Andreas Siegmund, Christina Bruss, Katja Dettmer, Peter J. Oefner, Linus Frick, Anna Weigert, Jonathan Jantsch, Wolfgang Herr, Michael Rehli, Kathrin Renner, Marina Kreutz

**Affiliations:** ^1^ Department of Internal Medicine III, University Hospital Regensburg, Regensburg, Germany; ^2^ Department for Otorhinolaryngology, University Hospital Regensburg, Regensburg, Germany; ^3^ Division of Interventional Immunology, Leibniz Institute for Immunotherapy (LIT), Regensburg, Germany; ^4^ Department of Plastic, Hand and Reconstructive Surgery, University Hospital Regensburg, Regensburg, Germany; ^5^ Department of Gynecology and Obstetrics, University Hospital Regensburg, Regensburg, Germany; ^6^ Institute of Functional Genomics, University of Regensburg, Regensburg, Germany; ^7^ Institute of Clinical Microbiology and Hygiene, University Hospital of Regensburg and University of Regensburg, Regensburg, Germany

**Keywords:** dendritic cells, glutamine, macrophages, glutamine synthetase, glutamate

## Abstract

**Introduction:**

Glutamine deficiency is a well-known feature of the tumor environment. Here we analyzed the impact of glutamine deprivation on human myeloid cell survival and function.

**Methods:**

Different types of myeloid cells were cultured in the absence or presence of glutamine and/or with L-methionine-S-sulfoximine (MSO), an irreversible glutamine synthetase (GS) inhibitor. GS expression was analyzed on mRNA and protein level. GS activity and the conversion of glutamate to glutamine by myeloid cells was followed by 13C tracing analyses.

**Results:**

The absence of extracellular glutamine only slightly affected postmitotic human monocyte to dendritic cell (DC) differentiation, function and survival. Similar results were obtained for monocyte-derived macrophages. In contrast, proliferation of the monocytic leukemia cell line THP-1 was significantly suppressed. While macrophages exhibited high constitutive GS expression, glutamine deprivation induced GS in DC and THP-1. Accordingly, proliferation of THP-1 was rescued by addition of the GS substrate glutamate and 13C tracing analyses revealed conversion of glutamate to glutamine. Supplementation with the GS inhibitor MSO reduced the survival of DC and macrophages and counteracted the proliferation rescue of THP-1 by glutamate.

**Discussion:**

Our results show that GS supports myeloid cell survival in a glutamine poor environment. Notably, in addition to suppressing proliferation and survival of tumor cells, the blockade of GS also targets immune cells such as DCs and macrophages.

## 1 Introduction

Even though glutamine is considered a non-essential amino acid, rapidly dividing cells such as enterocytes of the small intestine and many cancer cells have additional requirements for this amino acid, as it is needed for energy generation and as a source of carbon and nitrogen for biomass accumulation (1, 2).

Based on high glutamine consumption and catabolism, solid tumors often display glutamine deficiency relative to normal tissue and glutamine levels can drop to almost undetectable levels (3). Metabolomic analyses of freshly isolated human pancreatic ductal adenocarcinoma tumor specimens and benign adjacent tissue revealed that these tumors are low in glucose, glutamine and serine (4). Similar results were described by Sun et al. (5). Here, spatially resolved metabolomics showed that glutamine levels are (much) lower in cancer tissues from 256 esophageal squamous cell carcinoma patients compared with normal muscle and epithelial tissues. Instead, glutamate, the hydrolysis product of glutamine, was dramatically increased in cancer tissues. Lower levels of glutamine and higher levels of glutamate were also detected in a study including 75 breast cancer patients without metastases. Triple-negative breast cancer tumors contained lower levels of glutamine compared to triple-positive breast cancer tumors, which also indicates an accelerated glutaminolysis in this breast cancer subtype (6).

Auxotrophy, the dependency on external nutrient provision, is a well-known phenomenon not only for cancer cells but also for cells of the immune system depending on their supply with nonessential amino acids such as glutamine or asparagine. Therefore, glutamine availability has a huge influence on pathogen and cancer defense (7). Especially activated T cells seem to have an increased requirement for glutamine, leading to competition with tumor cells for this nutrient (8–10). Low glutamine levels in tumor tissues may therefore partially explain the lack of T cell infiltration. In line, Edwards et al. proposed a “glutamine steal” scenario, in which cancer cells limit glutamine availability for tumor-infiltrating lymphocytes, which in turn impairs the antitumor immune responses (10).

Besides T cells, tumor tissues are often highly infiltrated by myeloid cells, especially so-called tumor-associated macrophages (11), suggesting that glutamine metabolism differs between immune cell types and glutamine deficiency in the tumor environment may be less detrimental for myeloid cells (12). However, local glutamine deprivation by clear cell renal carcinoma tumor cells has an impact on myeloid activation as it induces IL-23 secretion by tumor-infiltrating macrophages (13), which might induce tumoricidal Th17 responses. Furthermore, inhibition of endogenous glutamine synthetase (GS, EC 6.3.1.2) activity in M2-like polarized tumor-promoting macrophages skews differentiation towards an M1-like phenotype (14). Even though the pro-inflammatory M1-like phenotype is regarded as anti-tumor macrophage and the anti-inflammatory M2-like state as tumor-promoting macrophage (15, 16), this is an oversimplified distinction as these cells are dynamic and heterogeneous within and across tumors. Their plasticity enables them to adapt to microenvironmental changes such as glutamine deficiency.

Up to now, glutamine metabolism and especially the influence of glutamine-deprivation on dendritic cell (DC) activation and differentiation is poorly explored. DCs act as immunological sentinels and play a critical role in the priming of local immunity but their functions are often suppressed in the tumor microenvironment resulting in DCs that promote immune tolerance (17, 18). For this reason, we investigated the influence of glutamine deficiency on human myeloid cells with a specific focus on DCs. Our data suggest that glutamine deficiency induces expression of GS in DCs and other myeloid cells and enables them to become independent from environmental glutamine supply.

## 2 Material and methods

### 2.1 Isolation and differentiation of monocyte-derived DCs and monocyte-derived macrophages

Peripheral blood mononuclear cells (PBMCs) of healthy donors were isolated by leukapheresis. Leukapheresis was approved by the Ethics committee of the University Hospital Regensburg (Ethic Vote Number 13-101-0240; 13-101-0238); all human participants gave written informed consent. Human monocytes were isolated from PBMCs by density gradient centrifugation over Ficoll/Hypaque followed by counterflow centrifugation elutriation (19). Monocytes were seeded on Teflon foils (to allow detachment of cells after cultivation, DuPont) at a concentration of 1×10^6^ cells/mL in RPMI1640 supplemented with 2% AB-serum (heat-inactivated; Bavarian red cross, Germany), penicillin (100 U/mL), streptomycin (100 µg/mL), with or without 2 mM L-glutamine (all from Thermo Fisher Scientific) with 2% pooled human serum for 7 days. Alternatively, macrophages were seeded at a concentration of 2×10^6^ cells per well in a 6-well plate in RPMI (supplements mentioned above). DCs were generated by incubating monocytes in RPMI1640 with 10% FCS (heat-inactivated; Sigma-Aldrich) and 224 IU/ml GM-CSF and 144 IU/ml IL-4 (both PeproTech) in the absence or presence of L‐glutamine for seven days. When indicated, cultures were treated with α-ketoglutarate (Keto; Sigma-Aldrich) at concentration of 1 mM or L-methionine-S-sulfoximine (MSO; Sigma-Aldrich) at a concentration of 1 mM.

### 2.2 Isolation and differentiation of murine bone-marrow derived macrophages

Bone marrow-derived macrophages (BMDM) were generated from bone marrow cells of wildtype C57BL/6NCrl (Charles River Breeding Laboratories) in Teflon bags containing DMEM medium (Gibco) supplemented with 10% fetal calf serum (FCS) (Sigma-Aldrich), 5% Horse Serum (Cell Concepts) and supernatant of L929 cells containing M-CSF for 7 days at 10% CO_2_ as described earlier (20, 21).

### 2.3 THP-1 culture

The human AML cell line THP-1 was obtained from the DSMZ (Braunschweig, Germany) and cultured in RPMI1640 (PAN, Aidenbach, Germany) supplemented with 10% fetal calf serum (FCS) (heat-inactivated), penicillin (50 IU/mL) and streptomycin (50 µg/mL), and 2 mM L-glutamine.

### 2.4 Determination of cell size and cell count

Cell proliferation in culture was monitored using the CASY Cell Counter system (OMNI Life Science).

### 2.5 Determination of cytokines

Cytokine secretion was determined in 24 h culture supernatants by commercially available enzyme-linked immunosorbent assays (R&D Systems) according to the manufacturer’s protocol.

### 2.6 [^13^C_5_]Glutamate tracer analysis

To perform [^13^C_5_]glutamate tracing in cell culture, human monocyte-derived dendritic cells, macrophages and the AML cell line THP-1 were cultured in glutamine rich media (as described above) for 7 days for primary human cells and 48 h for THP-1. Cells were then washed with glutamine-free medium and re-cultured for 24 h with 2 mM [^13^C_5_]glutamate (Cambridge Isotope Labs). After supernatants had been taken off, cells were washed twice in PBS and immediately frozen in liquid nitrogen. Supernatants and cell pellets were kept at −80°C until further analysis. Experiments were performed with cells from 3 different donors, for THP-1 at 3 different time-points each.

For metabolite extraction cell pellets were vortexed with 600 µL of 80% methanol (80:20 methanol: water, v/v) and centrifuged at 10.000 g and 4°C for 5 minutes. The supernatant was collected, and the remaining pellet was washed twice with 200 µL of 80% methanol employing 15.000 g for the last centrifugation step. The combined extracts were dried using an infrared vortex vacuum evaporator (CombiDancer, Hettich AG, Baech, Switzerland) and the residue was reconstituted in 100 µL pure water. Amino acids isotopologues were analyzed by high performance liquid chromatography (HPLC)–electrospray ionization (ESI)–tandem MS (MS/MS) after propyl chloroformate derivatization (22) employing a modified derivatization procedure that uses 17.4% propyl chloroformate and 82.6% isooctane as reagent 2. A 10 µL aliquot of the cell extract or 10 µL of cell culture supernatant were employed for derivatization. The MS/MS method was modified to include transitions for each possible isotopologue. Raw peak areas were corrected for natural stable isotope abundance and tracer impurity using IsoCorrectoR (23). Data are presented as mean ^13^C enrichment.

### 2.7 Western Blot analysis

Samples were lysed in RIPA (Sigma-Aldrich) separated by 12% SDS-PAGE and transferred to PVDF membranes, blocked with 5% milk (Sucofin) in TBS buffer with 0.1% Tween for 1 h, and incubated with primary antibodies overnight (GLUL; rabbit-anti-human/mouse, (Abcam Cat#197024, RRID: AB_10704544) and Actin; rabbit-anti-human/mouse, (Sigma-Aldrich Cat #A2066, RRID: AB_476693)). On the next day, membranes were washed and then incubated with the following secondary antibody for 1 h at RT: goat-anti-rabbit HRP (DakoCytomation Cat #P0448, RRID: AB_2617138). Detection was performed by chemiluminescence (ECL, Amersham Bioscience) and analyzed using the chemiluminescence system Fusion Pulse 6 (Vilber Lourmat).

### 2.8 Staining of extracellular surface markers and flow cytometry

In order to analyze immune cells, the following markers were used: CD11b FITC (BD Biosciences Cat #561015, RRID: AB_10561676), CD14 FITC (Beckman Coulter Cat# IM0645U, RRID : AB_130992), HLA-DR (BD Biosciences Cat #347400, RRID: AB_2868846), CD86 (BD Biosciences Cat #555657, RRID: AB_396012), HLA-ABC FITC (BD Biosciences Cat #555552, RRID: AB_395935), SIRP1α PE (BioLegend Cat #372104, RRID: AB_2650861), CD1a PE (Beckman Coulter, Cat #6603185), and DC-Sign PE (R and D Systems Cat #FAB161P, RRID: AB_357064). Cells were harvested in FACS tubes and washed with 1 ml of FACS wash buffer (PBS + 2% FCS). After addition of the antibodies, cells were incubated at 4°C for 20 min in the dark. After a washing step with 1 ml FACS wash buffer, cells were resuspended in FACS wash buffer and immediately analyzed by flow cytometry. To determine cell autofluorescence, unstained cells were used. Flow cytometry was performed using a BD FACS Calibur or Celesta. Data were analyzed with the FlowJo software (v10.8.1, Tree Star, RRID: SCR_008520).

### 2.9 Measurement of cellular oxygen consumption

Oxygen consumption was monitored non-invasively over time under cell culture conditions using the PreSens technology. 0.3×10^6^ cells were seeded in 1 ml in OD24 OxoDish^®^ plates (PreSens Precision Sensing GmbH) with pre-calibrated O_2_ sensors at the bottom of each well for non-contact reading by the SDR SensorDish^®^ Reader (PreSens Precision Sensing GmbH) through the transparent material of the plate.

### 2.10 Preparation of RNA, reverse transcription, and quantitative real-time PCR

Extraction of RNA, reverse transcription and quantitative real-time PCR for gene expression analysis in myeloid cells was performed as described elsewhere (24). Primer sequences (all from Eurofins MWG Operon, Ebersberg, Germany) were as follows (-5’-3’): 18S rRNA: sense, 5′-ACCGATTGGATGGTTTAGTGAG-3′ and antisense, 5′-CCTACGGAAACCTTGTTACGAC-3′. GLUL: sense, 5′-CATGTATCTCGTGCCTGCTGCC-3′ and antisense, 5′GTTGCTCACCATGTCCATTATCCGT-3′; GLUD1, gene-specific Primer PCR SYBR Green assay (Assay ID qHsaCED0038578) and GLUD2, gene-specific Primer PCR SYBR Green assay (Assay ID qHsaCED0038220).

### 2.11 siRNA mediated knockdown of glutamine synthetase

The siRNAs targeting GLUL and the respective non-target control pool were achieved from Dharmacon and dissolved in PCR-grade H_2_O to a final concentration of 1 µg/µL and stored at -20°C. Electroporation with siRNAs was performed with human monocyte-derived macrophages, which were differentiated for 7 days in RPMI1640 supplemented with 2% AB-serum, penicillin (100 U/mL), streptomycin (100 µg/mL), 2 mM L-glutamine and 2% pooled human serum. A total of 1×10^7^ macrophages per electroporation were washed first with RPMI1640 without phenol red and then with 10 mL OptiMEM (Thermo Fisher Scientific). Afterwards, cells were suspended in OptiMem to a final concentration to a final concentration of 1×10^7^ cells/mL. Electroporation with siRNA was performed with 1 µg siRNA/1×10^7^ cells using the Gene Pulser XCell Electroporation System (BioRad). Cells were directly seeded at a concentration of 1×10^6^ cells/mL in pre-warmed cell culture medium with or without 2 mM L‐glutamine.

### 2.12 Quantification and statistical analysis

Statistical parameters reported in the figures and the figure legends include the exact number of experiments (n), the definition of the center, statistical tests applied and statistical significance. Statistical analyses were performed with the GraphPad Prism software (version 9). Two-group comparisons of matched data sets were performed using the Wilcoxon test, as the number of experiments did not allow testing for normality distribution. Multiple comparisons of matched data sets were performed using the one-way ANOVA and post-hoc Tukey’s multiple comparison test or Friedman test and post-hoc Dunn’s or the two-way ANOVA and post-hoc Bonferroni’s multiple comparison test. In figures asterisks denote statistical significance (*p < 0.05, **p < 0.01, ***p < 0.001).

## 3 Results

### 3.1 Proliferation of monocytic THP-1 depends on exogenous glutamine supply

**Figure 1:**
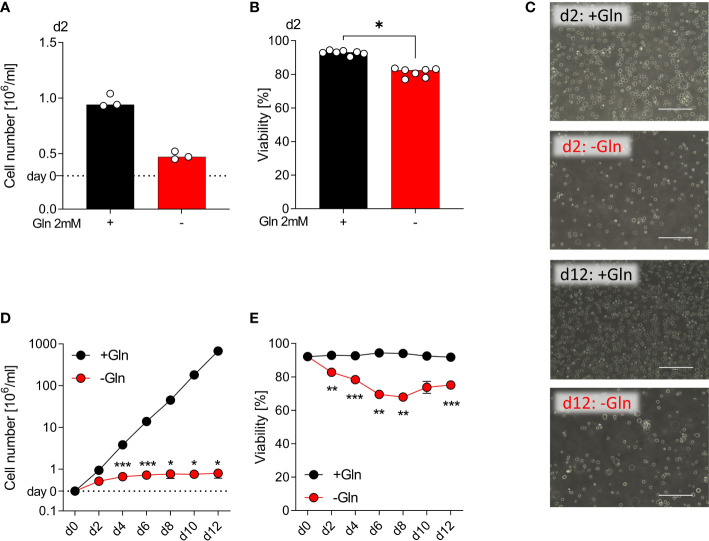
Proliferation of monocytic THP-1 depends on exogenous glutamine. THP-1 cells were cultured in the presence or absence of 2 mM glutamine (Gln). Cell yield **(A)** and cell viability **(B)** of human THP-1 cells were determined after 48 h using the CASY Cell Counter. Shown are median values and single data points. Statistical significance was calculated using Wilcoxon’s matched pair signed rank test (*p < 0.05). **(C)** Pictures taken on days 2 and 12 to document the morphology of THP‐1 cells; one representative picture is shown for each condition. Cell yield **(D)** and viability **(E)** of THP‐1 cells were analyzed every other day (mean ± SEM; n =3). Statistical significance was determined by two-way ANOVA and Bonferroni’s multiple comparison test (*p < 0.05; ***p < 0.001).

Myeloid cell lines can serve as a model system for human primary monocytes (25). Therefore, we investigated initially the impact of glutamine deficiency on the monocytic leukemia cell line THP-1. After 48 h of culture with glutamine (2 mM) and without glutamine (glutamine free medium plus serum), we determined cell numbers of THP-1 cells. Glutamine deprived culture conditions resulted in reduced proliferation (**Figure 1A**) but cell viability was preserved (**Figures 1B, C**). Next, we investigated the long-term impact of glutamine deprivation. THP-1 cells were counted and reseeded at 300.000 cells/mL every 48 h for 12 days and the theoretical cell yield was calculated. In the absence of glutamine, THP-1 cells showed almost no detectable proliferation, while in the presence of glutamine cells proliferated exponentially and showed consistently high viability (**Figures 1C–E**). These data indicate a strong dependence of THP-1 cells on exogenous glutamine supply, which might in part be related to their strong proliferative activity.

### 3.2 Differentiation of monocytes to dendritic cells is independent of extracellular glutamine

**Figure 2:**
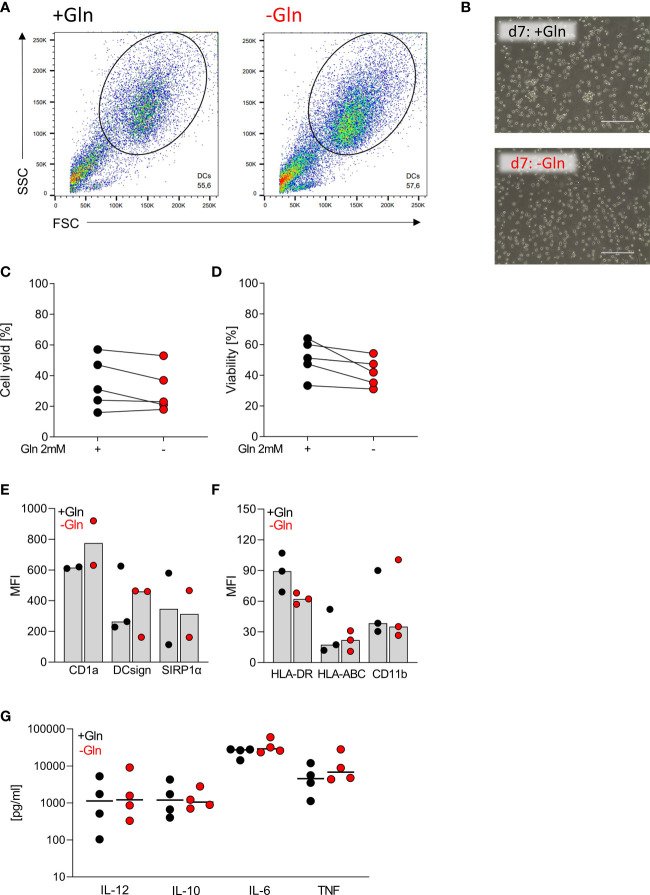
Differentiation of monocytes to dendritic cells is independent of exogenous glutamine. Human dendritic cells (DCs) were differentiated from monocytes in the presence of FCS, GM-CSF, and IL-4, with or without glutamine (Gln) for 7 days. **(A)** Representative gating of the differentiated DC population in SSC and FSC on day 7. **(B)** Pictures taken on day 7 to document the morphology of DCs; one representative picture is shown for each condition. **(C)** Cell count and **(D)** viability of differentiated DCs were determined on day 7 using the CASY Cell Counter. Symbols represent individual donors; lines link data points from the same donor. Statistical significance was calculated using Wilcoxon’s matched pair signed rank test (no significance detected). **(E, F)** Surface marker expression was analyzed by flow cytometry. Shown are median values and single data points. Statistical significance was calculated using Wilcoxon’s matched pair signed rank test (no significance detected). **(G)** Cytokines (IL-12, IL-10, IL-6, and TNF) were determined by ELISA in supernatants of DCs stimulated with LPS (100 ng/mL) for 24 h. Symbols represent individual donors. Statistical significance was calculated using Wilcoxon’s matched pair signed rank test (no significance detected).

Primary human monocytes, macrophages and DCs do not proliferate, and we were interested whether primary cells would, therefore, be less dependent on glutamine supply. We seeded monocytes in either glutamine-containing (2 mM) or glutamine-free medium in the presence of 10% FCS, GM-CSF and IL-4 to induce monocyte DC differentiation (26). Based on the literature 10% FCS should result in glutamine levels of approximately 0.1 mM in unsupplemented culture medium (27). Successful differentiation was followed by determining DC surface marker expression and LPS-stimulated cytokine secretion in culture supernatants. After 7 days, cell viability, cell numbers and morphology of monocyte-derived DCs were comparable (**Figures 2A–D**). The absence of glutamine did not affect the expression of DC surface markers such as CD1a, DC-SIGN, and HLA-DR (**Figures 2E, F**). Furthermore, production of IL-12, IL‐10, IL-6 and TNF was not altered by glutamine deprivation (**Figures 2G**).

### 3.3 Human macrophages do not rely on exogenous glutamine supply

**Figure 3:**
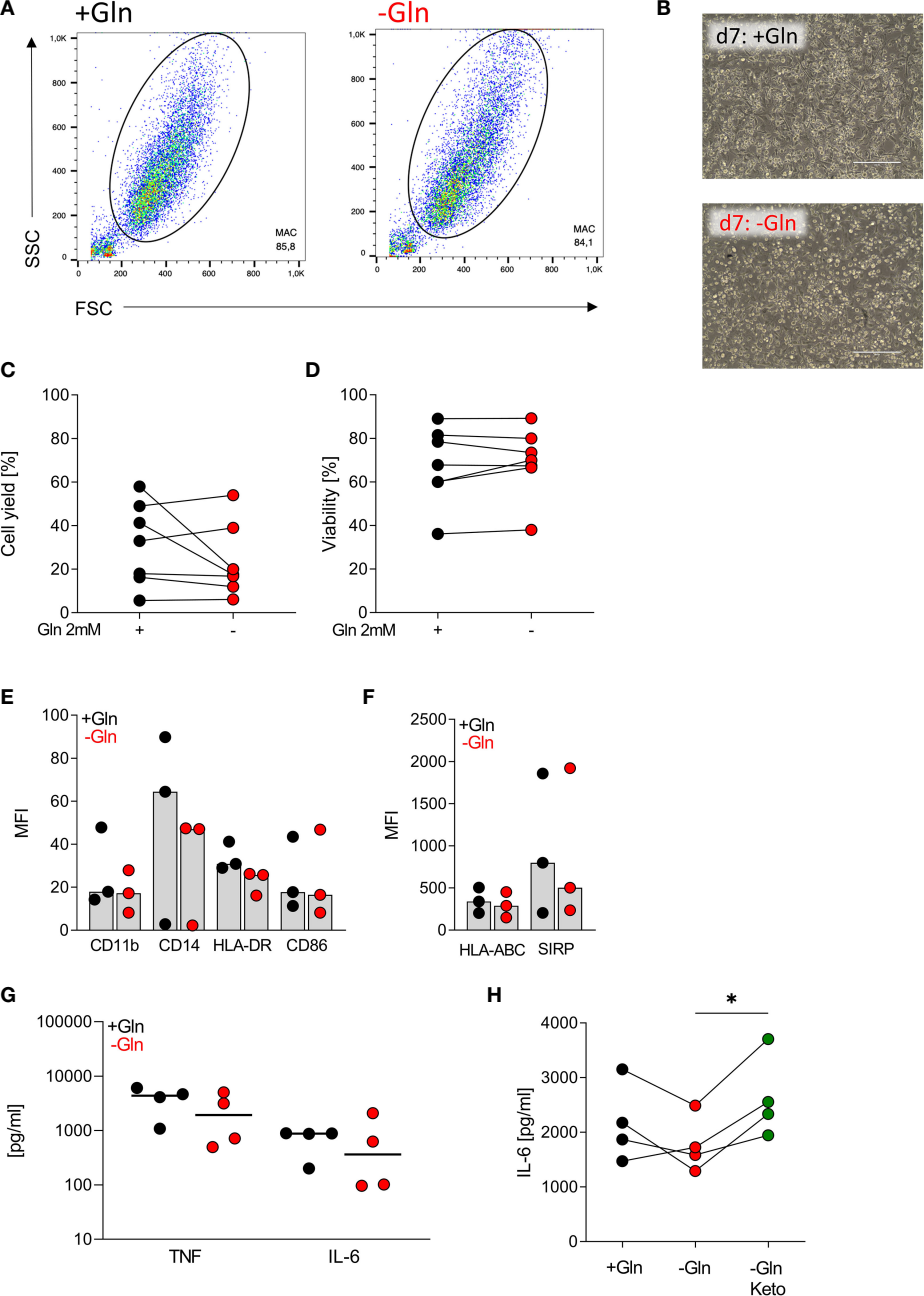
Human macrophages do not rely on exogenous glutamine. Human macrophages were differentiated from monocytes in the presence of human serum with or without glutamine (Gln) for 7 days. **(A)** Representative gating of the monocyte-derived macrophage population in SSC and FSC on day 7. **(B)** Pictures taken on day 7 to document macrophage morphology; one representative picture is shown for each condition. **(C)** Cell yield and **(D)** viability of differentiated macrophages were determined on day 7 using the CASY Cell Counter. Symbols represent individual donors; lines link data points from the same donor. Statistical significance was calculated using Wilcoxon’s matched pair signed rank test (no significance detected). **(E, F)** Surface marker expression was analyzed by flow cytometry. Shown are median values and single data points. Statistical significance was calculated using Wilcoxon’s matched pair signed rank test (no significance detected). **(G)** Cytokines (TNF and IL-6) were determined by ELISA in supernatants of macrophages stimulated with LPS (100 ng/mL) for 24 h. Symbols represent individual donors. Statistical significance was calculated using Wilcoxon’s matched pair signed rank test (no significance detected). **(H)** IL-6 was determined by ELISA in supernatants of macrophages stimulated with LPS (100 ng/mL) for 24 h. Symbols represent individual donors. Statistical significance was calculated using Friedman test with Dunn`s multiple comparison test (*p < 0.05).

Human blood monocytes can be differentiated into either DCs or monocyte-derived macrophages *in vitro*. To study the impact of glutamine on differentiation and activation of monocyte-derived macrophages, we cultured monocytes on Teflon foils for 7 days with 2% human serum with and without glutamine. 2% human serum should result in about 0.012 mM glutamine in supplement-free culture medium as human serum contains ~ 0.6 mM glutamine (28–30). Similar to our results with DCs, no major differences were observed regarding cell viability, cell yield and morphology (**Figures 3A–D**). The expression of various surface markers as well as LPS-stimulated cytokine secretion was also not significantly altered (**Figures 3E–G**). We also investigated the impact of glutamine shortage in a short-term setting. Here, by trend IL-6 secretion was lowered and addition of α-ketoglutarate (Keto) partially counteracted the effect (**Figure 3H**).

Furthermore, we investigated whether macrophage polarization and different culture conditions would change glutamine dependency. Monocytes were differentiated into macrophages on plastic plates in the presence of 2% human serum with or without IL-10 or LPS. Again, glutamine deprivation did not lead to macrophage cell death and did not block differentiation-associated morphological changes (**Figures S1A–C**).

### 3.4 THP-1 adapt to glutamine-free conditions by upregulation of glutamine synthetase

**Figure 4:**
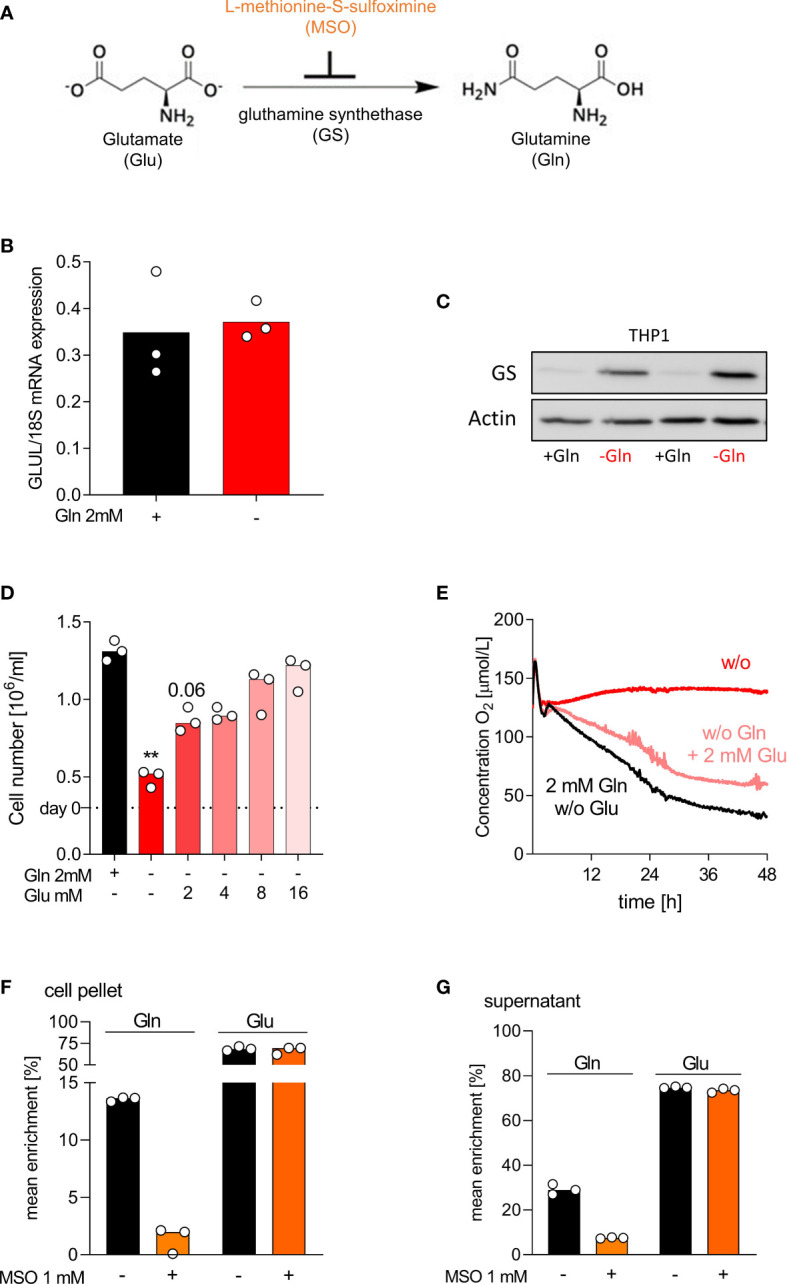
THP-1 adapt to glutamine-free conditions by upregulation of glutamine synthetase. **(A)** Schematic representation of Gln neogenesis; Glutamate (Glu) can be amidated to Gln by glutamine synthetase (GS). The enzyme GS is irreversibly inhibited by the substance L-methionine-S-sulfoximine (MSO). **(B, C)** THP-1 cells were cultured in presence or absence of 2 mM glutamine (Gln). **(B)** The mRNA expression of GLUL was determined by qPCR after 2 days and normalized to 18S mRNA. Shown are median values and single data points. Statistical significance was calculated using Wilcoxon’s matched pair signed rank test (no significance detected). **(C)** Western Blot analysis of GS in THP-1 after 2 days. **(D)** Cell yield of THP-1 was analyzed after 2 days of culture with or without 2 mM Gln or increasing Glu concentrations (2 mM, 4 mM, 8 mM, 16 mM). Shown are median values and single data points. Statistical significance was calculated using Friedman test with Dunn`s multiple comparison test (**p < 0.01). **(E)** Oxygen consumption was monitored in the absence (w/o, without) or presence of 2 mM Gln or 2 mM Glu using the PreSens technology (mean values, n = 3). **(F, G)** THP‐1 cells were incubated with 2 mM [^13^C_5_]glutamate in the presence or absence of 1 mM MSO for 24 h. After 24 h, cells were lysed and the mean enrichment of ^13^C in glutamate and glutamine were determined in cell pellets **(F)** and supernatants **(G)** by mass spectrometry. Shown are median values and single data points. Statistical significance was calculated using Wilcoxon’s matched pair signed rank test (no significance detected).

Protein expression of glutamine synthetase (GS), the enzyme that catalyzes *de novo* glutamine synthesis, largely determines glutamine dependency. GS expression varies between cells and is low in auxotrophic (tumor) cells, which leads to glutamine-addiction (31). The conversion of glutamate, ATP and ammonia into glutamine is irreversibly blocked by L-methionine-S-sulfoximine (MSO) (32) (schematic view **Figure 4A**). To investigate the observed differences in glutamine dependency, we measured the mRNA expression of glutamine synthetase (*GLUL*), glutaminase (*GLS*) and glutamate dehydrogenase (*GLUD1*) by RT-PCR in THP-1 and human macrophages grown in glutamine-rich (2 mM) and glutamine-poor medium. *GLUL* mRNA expression was detected in THP-1 and not altered by glutamine absence or presence (**Figure 4B**). In contrast, GS protein expression was low in the presence of glutamine and strongly upregulated under glutamine deprivation (**Figure 4C**). Macrophages showed a strong constitutive expression of *GLUL* mRNA and GS protein, which was by trend slightly upregulated in the absence of glutamine (**Figures S2A, B**). Surprisingly, GS protein was almost undetectable in murine macrophages, which may also relate to the different culture conditions between human and murine macrophages (**Figure S2B**). Glutaminase mRNA expression was lower in primary cells, while *GLUD1* expression did not differ between leukemia cells and macrophages (**Figures S2C, D**).

As THP-1 cells upregulated GS under glutamine-deficient culture conditions, we wondered whether glutamate supplementation could rescue THP-1 proliferation. Indeed, glutamate increased dose-dependently the proliferation of THP-1 cells in absence of glutamine and cell counts returned to control levels upon high glutamate supplementation (**Figure 4D**). Glutamate also reverted the suppression of oxygen consumption in THP-1 cells (**Figure 4E**). Additional MSO treatment reversed the positive effect of glutamate, whereas cells cultured in the presence of glutamine were not affected by MSO (data not shown). To determine the enzyme activity of GS in THP-1, we performed tracing analyses. THP‐1 cells were incubated for 24 h with 2 mM [^13^C]glutamate in the presence or absence of 1 mM MSO. The intracellular and extracellular levels of [^13^C]glutamine and [^13^C]glutamate, resulting from intracellular glutamate metabolism, were determined in cell lysates and supernatants of THP-1 cells by mass spectrometry. As expected, glutamate supplementation resulted in intracellular and extracellular glutamine ^13^C enrichment, which was decreased by MSO treatment (**Figures 4F, G**). Higher enrichment was detected in macrophages (**Figures S2E, F**). Surprisingly MSO did only partially suppress glutamine production in macrophages.

### 3.5 GS inhibition by MSO impedes the differentiation of monocytes into DCs and macrophages

**Figure 5:**
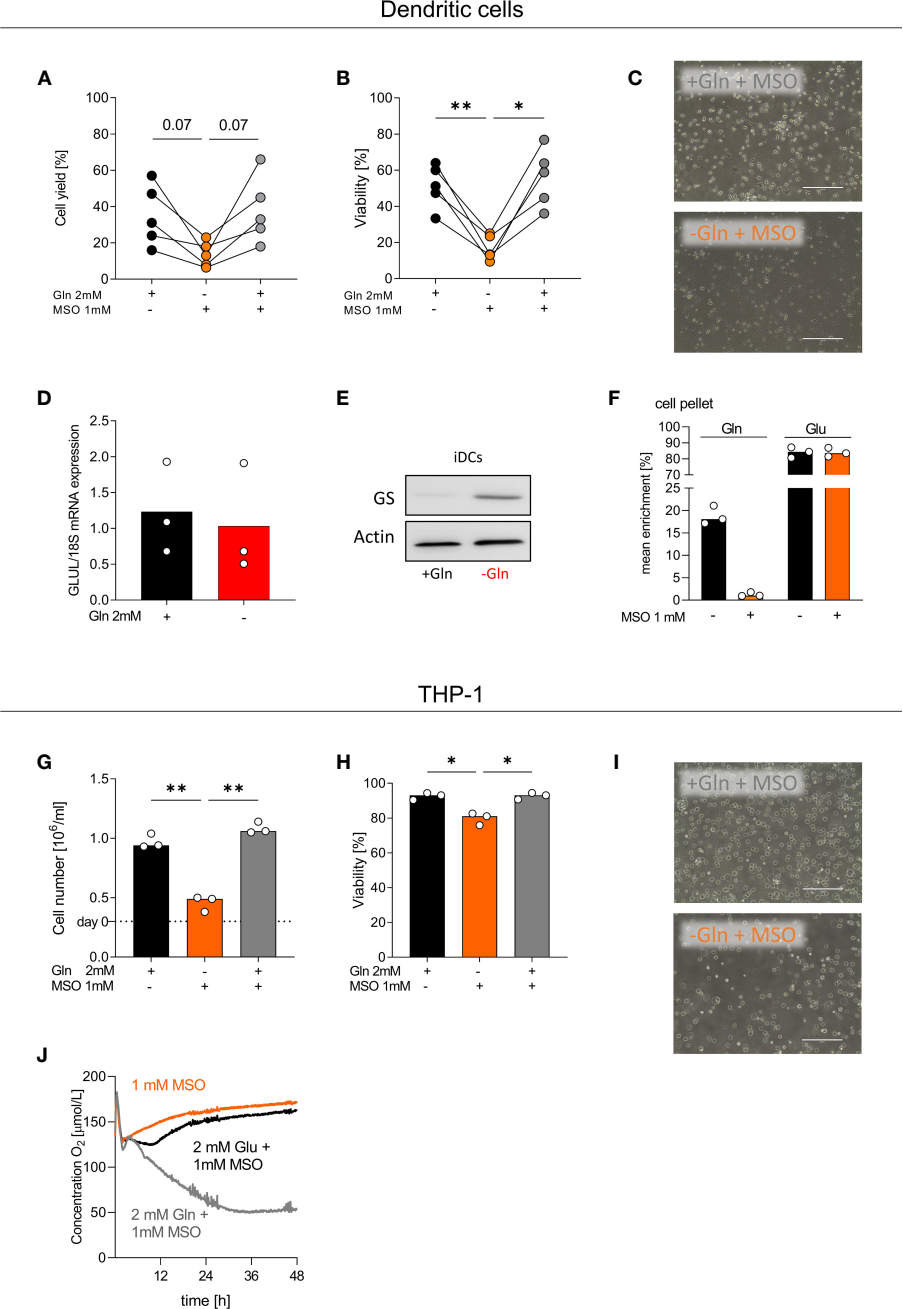
GS inhibition by MSO impedes the differentiation of monocytes into DCs. **(A-F)** Human dendritic cells (DCs) were differentiated from monocytes in the presence of FCS, GM-CSF, IL-4, and with or without 2 mM glutamine (Gln) and/or 1 mM MSO for 7 days. **(A)** Cell count and **(B)** viability of differentiated DCs were determined on day 7 using the CASY Cell Counter. Symbols represent individual donors; lines link data points from the same donor. Statistical significance was calculated using one-way ANOVA and Tukey’s multiple comparison test (*p < 0.05, **p < 0.01). **(C)** On day 7, pictures were taken to document the morphology of DCs; one representative picture is shown for each condition. **(D)** The mRNA expression of GLUL by DCs was determined by qPCR and normalized to 18S mRNA. Statistical significance was calculated using Wilcoxon’s matched pair signed rank test (no significance detected). **(E)** Western Blot analysis of glutamine synthetase (GS) in DCs. **(F)** DCs were incubated with 2 mM [^13^C_5_]glutamate in the presence or absence of 1 mM MSO for 24 h. After 24 h, cells were lysed and mean enrichment of ^13^C glutamate and glutamine was determined in cell pellets. Shown are median values and single data points. Statistical significance was calculated using Wilcoxon’s matched pair signed rank test (no significance detected). **(G-J)** THP-1 cells were cultured in the presence or absence of 2 mM glutamine (Gln) and/or 1 mM MSO. Cell yield **(G)** and cell viability **(H)** of human THP-1 cells were analyzed after 48 h. Shown are median values and single data points. Statistical significance was calculated using one-way ANOVA and Tukey’s multiple comparison test (*p < 0.05, **p < 0.01). **(I)** After 48 h, pictures were taken to document the morphology of THP-1 cells; one representative picture is shown for each condition. **(J)** Oxygen consumption was monitored in the absence or presence of 2 mM Gln or 2 mM Glu and/or 1 mM MSO using the PreSens technology (mean values, n = 3).

To explore the impact of GS on monocyte differentiation, we cultured monocytes with or without glutamine in the presence or absence of MSO, blocking GS activity. MSO treatment had no impact on DC viability or cell yield in the presence of glutamine but severely affected survival in the absence of glutamine (**Figures 5A–C**). GS was strongly upregulated in DCs cultured without glutamine, notably on protein but not on mRNA level (**Figures 5D, E**). DCs metabolized glutamate to glutamine, which was completely blocked by MSO (**Figure 5F**). MSO also impaired viability, growth and respiration of THP-1 cells under glutamine-restricted culture conditions (**Figures 5G–J**). In contrast, MSO had no effect on macrophage survival (**Figure S3A–C**) in line with our finding that MSO did not completely block GS activity in macrophages. Therefore, we analyzed whether targeting of GLUL protein directly with siRNA would alter macrophage differentiation. In 4 experiments knockdown of GLUL slightly lowered the cell yield in the absence but not in the presence of glutamine (**Figure S3D**). These results demonstrate that GS is essential for myeloid cells under conditions of glutamine shortage, which might be especially important in the tumor environment.

## 4 Discussion

Glutamine provides intermediates for many metabolic pathways and is especially important for proliferating cells such as tumor cells but also activated immune cells. Based on the strong demand, increased glutamine catabolism may lead to glutamine deprivation and competition for glutamine, especially in the core region of solid tumors, which often display glutamine deficiency compared with other amino acids (3). Glutamine addiction is also a hallmark of clear cell renal cell carcinoma (33). But glutamine dependency and glutamine levels often relate to glutamine synthetase (GS) expression which varies greatly between tumors, ranging from negative to high. In principle, GS-positive tumor cells can autonomously synthesize glutamine from glutamate, whereas GS-negative tumor cells need exogenous supply. In glioblastoma tumors, glutamine required for tumor growth can be autonomously synthesized by GS-positive glioma cells (34), whereas downregulation of GS is responsible for glutamine addiction in acute lymphoblastic leukemia (31).

Here we found that THP-1 AML tumor cells but also immune cells can express and upregulate GS expression under metabolic pressure. THP-1 tumor cells rely on glutamine supply under normal culture conditions. However, under glutamine deprivation THP-1 tumor cells upregulate GS expression on the protein level, which enables them to produce glutamine from glutamate. This in turn rescues cell respiration and proliferation in the presence of glutamate. GS mRNA was not upregulated indicating a posttranscriptional mechanism of GS regulation. The work by Labow et al. suggests, that glutamine levels regulate the stability of GS. High glutamine levels lead to enhanced GS degradation, whereas low glutamine levels increase GS stability (35). Furthermore, Arad et al. and Crook et al. described that GS is subject to feedback control by glutamine, which promotes its degradation (36, 37). Thang Van Nguyen and colleagues reported a potential molecular basis for this regulation (38). High glutamine concentrations lead to GS lysine acetylation, resulting in binding and ubiquitylation by cereblon CRL4CRBN and in turn degradation, i.e. acetylation adjusts GS protein levels in response to glutamine (38).

Similar to THP-1 cells, GS was not expressed in the presence of glutamine in monocyte-derived dendritic cells but strongly induced under glutamine deprivation (glutamine free media were supplemented with serum resulting in very low glutamine levels). Therefore, it seems that a glutamine-rich microenvironment alleviates the need to synthesize glutamine, but deprivation enforces GS expression and stability, respectively. Up to now, little is known regarding glutamine metabolism during DC activation and differentiation. Glutamine is important for TLR-induced activation of human plasmacytoid DCs but TLR-stimulation induces glycolysis in CD1c^+^ mDC (39).

Glutamine metabolism differs between different types of immune cells and is determined by the activation and differentiation status of the respective cell type (40). In contrast to myeloid tumor cells and DCs, macrophages showed a high constitutive GS expression. Accordingly, the Mazzone group described, that resting human macrophages express high GS protein levels (14). In their experiments IL-10-stimulated human M2-like macrophages showed even higher GS expression and inhibition of GS activity in M2-like macrophages with the GS inhibitor MSO skewed their polarization towards an M1 state, even though glutamine was still present in the medium. Interestingly, our data show that GS protein expression is almost undetectable in murine bone-marrow derived macrophages, independent of M1- or M2-like culture conditions, indicating species dependent differences in GS regulation. In mice, GS expression seems to be relevant for the tumor-promoting function of tumor-associated macrophages as genetic deletion of GS in macrophages inhibited metastasis in tumor bearing mice (14). Moreover, animal experiments showed that glutamine is essential for the production of cytokines, antigen presentation, and phagocytic functions in murine macrophages (41). Others have shown that especially murine M2-like macrophages consume glutamine as production of α-ketoglutarate *via* glutaminolysis is important for alternative M2-like activation (42).

In our study glutamine deprivation itself did not alter cytokine secretion or surface antigen expression neither in human monocyte-derived DC nor in macrophages. In contrast, Spittler and colleagues demonstrated that glutamine is indispensable for the expression of immune response-associated surface markers of myeloid leukocytes (43). However, we cannot exclude that MSO treatment would have more impact on myeloid differentiation as GS might play a cell intrinsic role for macrophage and/or DC differentiation independent of extracellular glutamine supply.

In line with Palmieri et al. (14), starvation with very low glutamine in the culture medium tended to enhance GS expression in macrophages, in both M0 and IL-10-treated macrophages. More interestingly, culturing macrophages and dendritic cells with very low glutamine levels in the presence of glutamate promoted glutamate uptake and conversion to glutamine. Glutamine was not only produced but also secreted, as it was detected in supernatants. MSO suppressed GS activity and thereby glutamine production and secretion in DCs but surprisingly macrophages still continued to produce glutamine suggesting that that macrophages might be endowed with other enzymes that allow glutamine production in the presence of MSO. Alternatively, the MSO dose applied could be too low as GS expression was very high in macrophages.

Overall, GS expression in myeloid cells (which was enhanced under starved conditions) might promote glutamine secretion for use by other cells. Similar to myeloid cells, glutamine synthesis is up-regulated in cancer-associated fibroblasts and accompanied by glutamine secretion to rescue cancer cell growth in a glutamine-deficient tumor environment. Abrogation of glutamine anabolism in fibroblasts can inhibit ovarian tumor growth in mice (44).

Strong (constitutive) GS expression and glutamine secretion could play an important role in physiologic glutamine homeostasis as glutamine restriction is not only a phenomenon described for the tumor environment. Under steady state conditions, the muscle tissue is a major site for glutamine synthesis but muscle injuries and ageing can lead to intra-tissue glutamine restriction. In a mouse model, muscle-infiltrating macrophages sense glutamine shortage and start to secrete glutamine (45).

Yet, whether local glutamine metabolism impacts immune surveillance is less investigated. Glutamine is important for proliferating cells such as tumor cells or activated T cells (8), but surprisingly adoptive transfer of tumor-specific CD8^+^T cells cultured under glutamine-restricted conditions or treated with specific inhibitors of glutamine metabolism even promoted tumor control (46). In line, glutamine blockade in tumor bearing mice suppressed metabolism of cancer cells and supported effector T cells (47) and in combination with anti-PD-L1, inhibition of glutamine metabolism promoted the antitumor efficacy of T cells (48). These data suggest that glutamine addiction of T cells might relate to specific T cell subtypes and/or culture conditions *in vitro.*

The Haigis group has shown that accelerated glutamine catabolism by tumor cells leads to accumulation of increased amounts of ammonia in the tumor environment that can be recycled to glutamate (49). Tumor-associated myeloid cells and/or fibroblasts may take advantage of this and further metabolize glutamate to glutamine – a perfect metabolic symbiosis where (myeloid) stromal cells maintain cancer cell growth when glutamine is scarce.

In summary, myeloid GS expression could play an important role under physiological and pathophysiological conditions. Targeting the metabolic crosstalk between myeloid cells, such as macrophages and DCs, and tumor cells represents an interesting anti-tumor strategy.

## Data availability statement

The original contributions presented in the study are included in the article/**Supplementary Material**, further inquiries can be directed to the corresponding authors.

## Ethics statement

The studies involving human participants were reviewed and approved by Ethics committee of the University Hospital Regensburg, Regensburg, Germany. The patients/participants provided their written informed consent to participate in this study.

## Author contributions

Conceptualization: MK. Methodology: KD, PO. Formal analysis: KD, PO. Investigation: RS, NB, SM-D, CB, AS, LF, AW, JJ, MR. Resources: WH. Writing/original draft preparation: MK, RS, NB, KR. Writing/review and editing: MK, NB, KR. Supervision: MK and KR. All authors have read and agreed to the published version of the manuscript. All authors contributed to the article and approved the submitted version.
