# Supplementary material for: Glutamine synthetase expression rescues human dendritic cell survival in a glutamine-deprived environment
Source: Front Oncol. 2023 Jan 19;13:1120194. doi: 10.3389/fonc.2023.1120194 (PMC9894315; doi:10.3389/fonc.2023.1120194)
Supplement: Supplementary file 1 [file DataSheet_1.pdf]

Figure S1

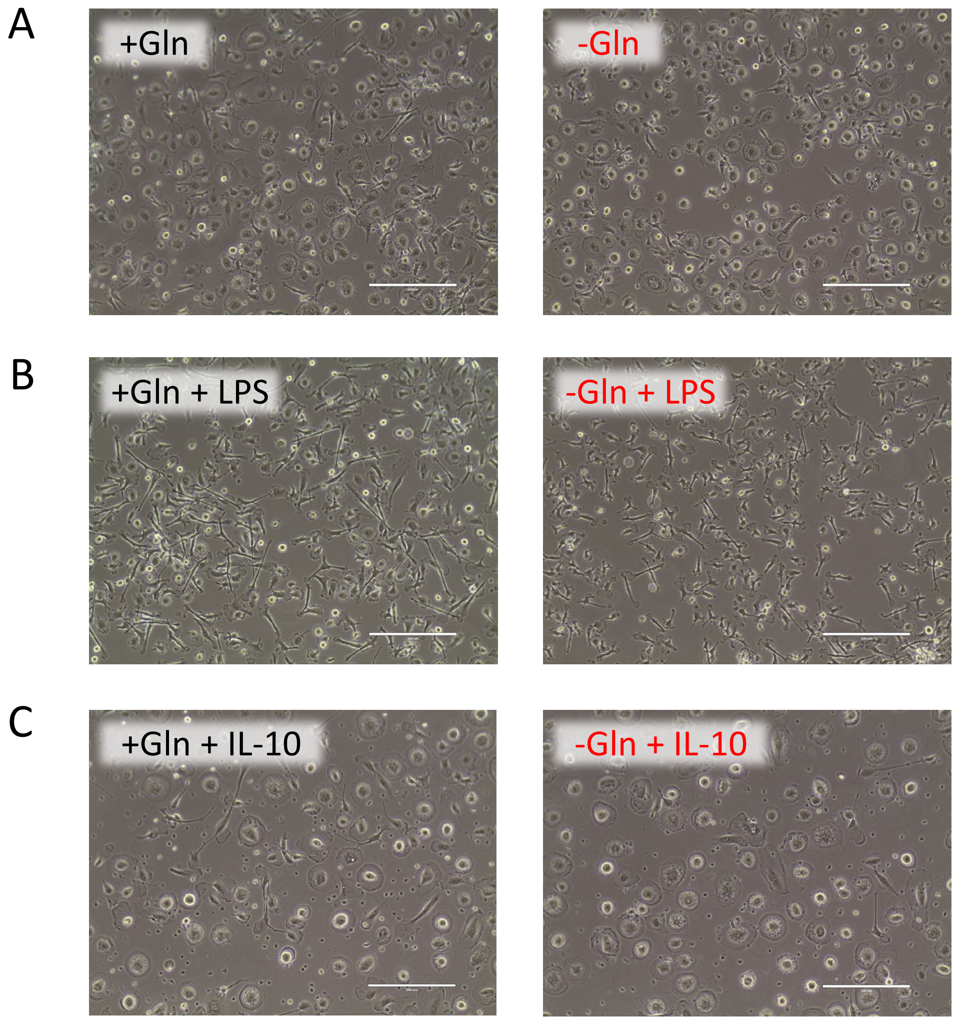

Figure S1. Glutamine depletion has no impact on morphology of macrophages. Human macrophages were differentiated from monocytes in presence of human serum with or without glutamine (Gln) for 7 days. Monocyte-derived macrophages were treated without an additional stimulus (**A**), with LPS (100 ng/mL) (**B**) or IL-10 (**C**). Pictures were taken to document the morphology of macrophages; one representative picture is shown for each condition.

Figure S2

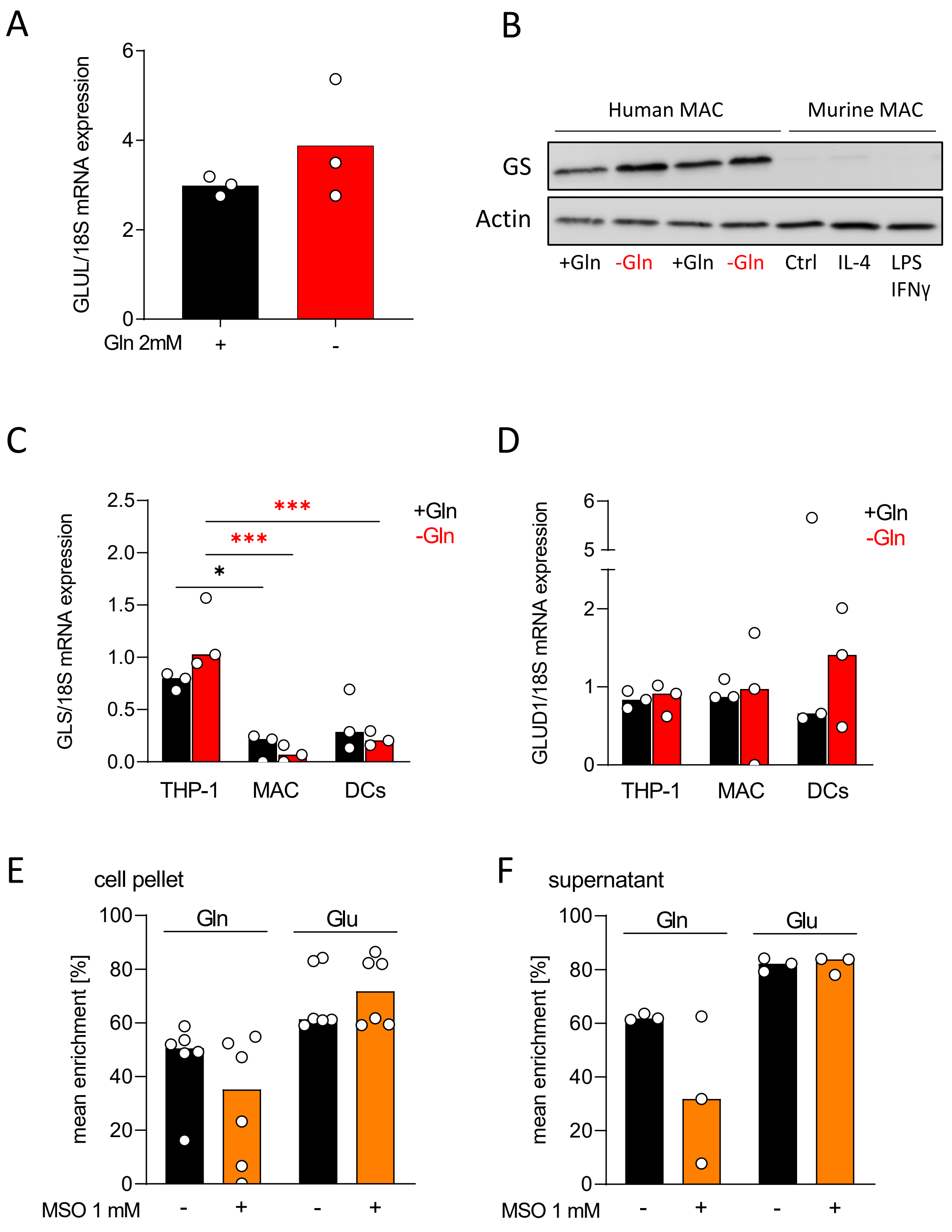

Figure S2. Human but not murine macrophages express glutamine synthetase. (**A-F**) Human macrophages were differentiated from monocytes in the presence of human serum with or without glutamine (Gln) for 7 days. (**A**) Gene expression of f glutamine synthetase (GLUL) was determined by qPCR and normalized to 18S mRNA. Data are show as mean, n = 3 different donors. Statistical significance was calculated using Wilcoxon's matched pair signed rank test (no significance detected). (**B**) Western Blot analysis of glutamine synthetase (GS) in human monocyte-derived macrophages and murine bone-marrow derived macrophages with or without IL 4 (10 ng/mL) or LPS (10 ng/mL) and IFNγ (20 ng/mL). (**C,D**) mRNA expression of glutaminase (GLS, **C**) and glutamate dehydrogenase (GLUD1, **D**) from THP-1, macrophages (MAC) and dendritic cells (DCs) was determined by qPCR and normalized to 18S mRNA. Shown are median values and single data points. Statistical significance was calculated using one-way ANOVA and Tukey's multiple comparison test (*$p < 0.05$, ***$p < 0.001$). **(E,F)** Macrophages were incubated with 2 mM [$^{13}C_5$]glutamate in the presence or absence of 1 mM MSO for 24 h. After 24 h, cells were lysed and mean enrichment of ^{13}C in glutamate and glutamine was determined in cell pellets (**E**) and supernatants (**F**) by mass spectrometry. Shown are median values and single data points. Statistical significance was calculated using Wilcoxon's matched pair signed rank test (no significance detected).

Figure S3

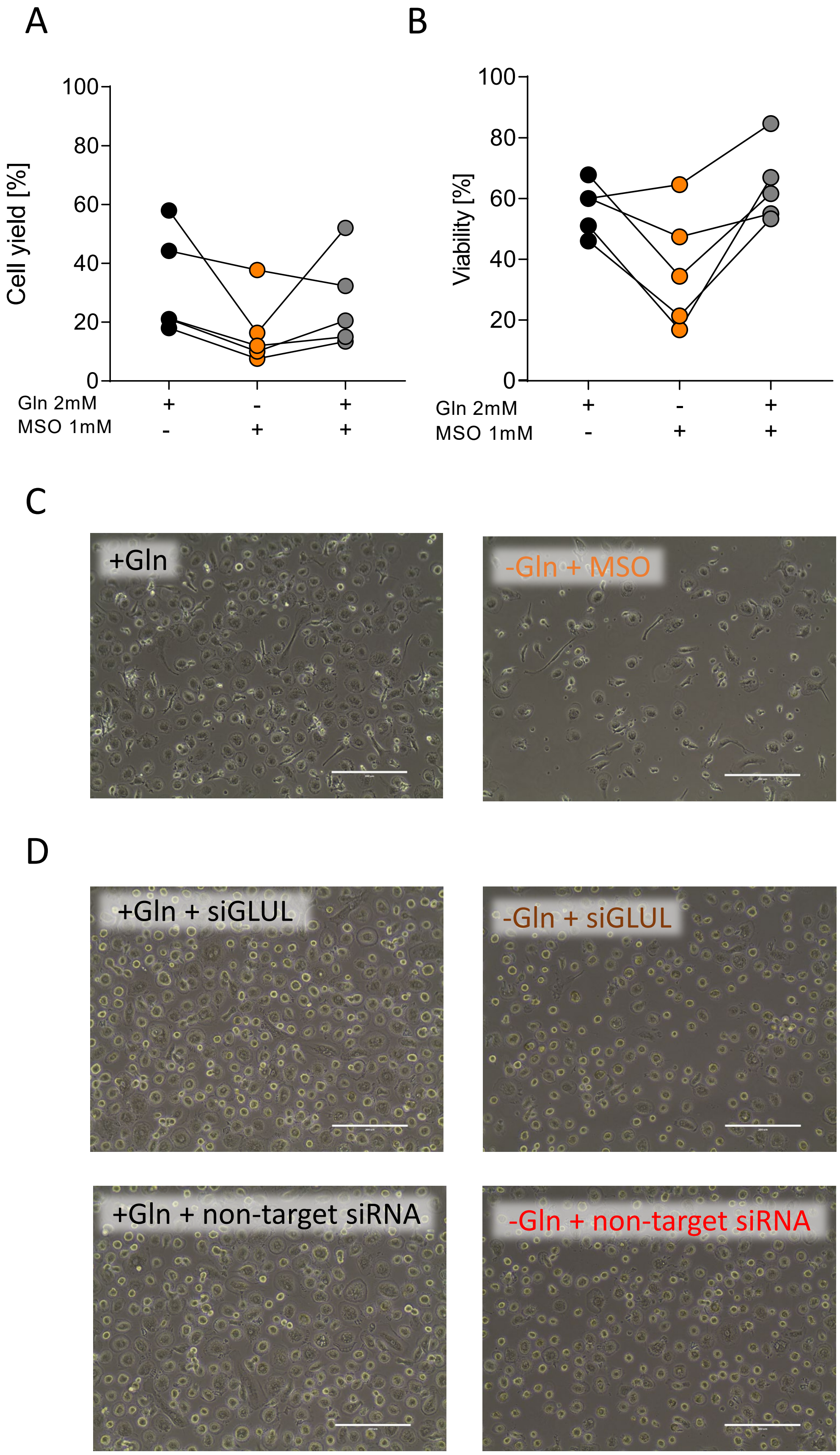

Figure S3. Impact of GS inhibition by MSO on human macrophages. (**A-C**) Human macrophages were differentiated from monocytes in the presence of human serum with or without 2 mM glutamine (Gln) and/or 1 mM MSO for 7 days. (**A**) Cell yield and (**B**) viability of differentiated macrophages were determined on day 7 using the CASY Cell Counter. Symbols represent individual donors; lines link data from same donor. Statistical significance was calculated using one-way ANOVA and Tukey's multiple comparison test (no significance detected. (**C**) Pictures taken on day 7 to document the morphology of macrophages; one representative picture is shown for each condition. (**D**) Human monocyte-derived macrophages were electroporated with siRNA targeting GLUL (siGLUL) or a non-control (non-target siRNA) cultured with or without 2 mM Gln. Pictures taken on day 7 to document the morphology of macrophages; one representative picture of four experiments is shown for each condition.
